# Gene amplification of EGFR, HER2, FGFR2 and MET in esophageal squamous cell carcinoma

**DOI:** 10.3892/ijo.2013.1830

**Published:** 2013-02-19

**Authors:** HIROAKI KATO, TOKUZO ARAO, KAZUKO MATSUMOTO, YOSHIHIKO FUJITA, HIDEHARU KIMURA, HIDETOSHI HAYASHI, KOUHEI NISHIKI, MITSURU IWAMA, OSAMU SHIRAISHI, ATSUSHI YASUDA, MASAYUKI SHINKAI, MOTOHIRO IMANO, HARUHIKO IMAMOTO, TAKUSHI YASUDA, KIYOTAKA OKUNO, HITOSHI SHIOZAKI, KAZUTO NISHIO

**Affiliations:** 1 Departments of Surgery, Kinki University Faculty of Medicine, Osaka 589-8511, Japan; 2 Genome Biology, Kinki University Faculty of Medicine, Osaka 589-8511, Japan

**Keywords:** EGFR, HER2, FGFR2, MET, esophageal squamous cell carcinoma

## Abstract

Molecular targeted therapy is expected to be a promising therapeutic approach for the treatment of esophageal squamous cell carcinoma (ESCC); however, the gene amplification status of molecular targeted genes in ESCC remains largely unclear. The gene amplification of *EGFR, HER2, FGFR2* and *MET* was examined using a real-time PCR-based copy number assay of 245 ESCC surgical specimens of formalin-fixed, paraffin-embedded samples. Fluorescence *in situ* hybridization (FISH) and comparative genomic hybridization analyses verified the results of the copy number assay. *EGFR* mutation was detected using the Scorpions-ARMS method. The *EGFR* status and drug sensitivity to an EGFR tyrosine kinase inhibitor was then evaluated *in vitro*. Gene amplification of *EGFR* and *HER2* was observed in 7% (16/244) and 11% (27/245) of the ESCC specimens. A multivariate analysis revealed that *HER2* amplification was a significant predictor of a poor prognosis in patients with stage III post-operative ESCC. The L861Q type of *EGFR* mutation with hypersensitivity to EGFR tyrosine kinase inhibitor was found in one of the eight ESCC cell lines and one del745 type of *EGFR* mutation was identified in 107 clinical samples. In addition, we demonstrated for the first time that *FGFR2* amplification was observed in 4% (8/196) of the ESCC specimens. *MET* amplification was observed in 1% (2/196). In conclusion, the frequent gene amplification of *EGFR*, *HER2* and *FGFR2* and the presence of active *EGFR* mutations were observed in ESCC specimens. Our results strongly encourage the development of molecular targeted therapy for ESCC.

## Introduction

Despite extensive investigations of therapeutic improvements in surgical techniques, chemotherapy and chemo-radiotherapy, esophageal squamous cell carcinoma (ESCC) remains one of the most aggressive and fatal malignancies and the prognosis of patients with ESCC remains poor (1). Although curative surgical resection can be performed, half of all patients develop recurrences within a few years after surgery and the 5-year survival rate is only approximately 50% (2). Therefore, more effective therapies are urgently needed to improve the prognosis of patients with ESCC.

The overexpression of epidermal growth factor receptor (EGFR) and HER2 can be observed in a variety of human malignancies and the roles of such overexpressions in cancer development, progression and aggressiveness have been widely recognized (3,4). Approximately 50–70% of ESCC tumors express *EGFR* protein when examined using immunohisto-chemistry (IHC), while 15–28% of specimens exhibit *EGFR* gene amplification when examined using fluorescence *in situ* hybridization (FISH) (5,6). Similarly, HER2 protein overexpression has been observed in 30–41% of specimens examined using IHC, while *HER2* gene amplification has been observed in 11–19% of specimens using FISH (7–9). These results indicate that EGFR and HER2 overexpression and gene amplification are frequently observed in ESCC, strongly suggesting that signaling involving these factors may play important biological roles and may be useful molecular targets in ESCC. Somatic mutations of EGFR tyrosine kinase in non-small cell lung cancer (NSCLC) have been shown to increase kinase activity and to be associated with hypersensitivity to gefitinib, a selective EGFR tyrosine kinase inhibitor (EGFR-TKI) (10,11). A recent phase III study demonstrated that first-line gefitinib for patients with advanced NSCLC with *EGFR* mutations improved progression-free survival, compared with standard chemotherapy (12). Therapeutics targeting EGFR and HER2, such as small-molecule inhibitors or specific monoclonal antibodies, are now under intensive investigation in clinical settings and some of them have achieved clinical success in the treatment of diverse solid cancers (4,13).

Fibroblast growth factor receptor (FGFR) signaling is deregulated in a wide variety of cancers (14). We previously reported that *FGFR2* amplification was observed in 4.1% of gastric cancers and that *FGFR2* amplification confers hypersensitivity to FGFR inhibitor in gastric cancer cell lines both *in vitro* and *in vivo*(15,16), strongly suggesting that *FGFR2* amplification may be a promising molecular target for gastric cancer treatment. In ESCC, information regarding *FGFR2* amplification remains unclear. Additionally, hepatocyte growth factor (HGF)-MET receptor signaling provides important signals for cell survival and migration in cancer cells; thus, these molecules have also emerged as promising molecular targets for cancer therapy (17).

Very limited information is available regarding the gene amplification of *EGFR, HER2, FGFR2* and *MET* and the *EGFR* mutation status in relation to the prognostic impact for post-curative surgery in ESCC. In an attempt to advance molecular-targeted therapy for ESCC, we retrospectively studied these issues using formalin-fixed, paraffin-embedded (FFPE) samples from patients with ESCC who had undergone surgery.

## Materials and methods

### Cell culture

KYSE170, KYSE180 and KYSE270 were maintained in a 1:1 mixture of Ham’s F12 medium and RPMI-1640 medium (Sigma, St. Louis, MO) supplemented with 2% heat-inactivated fetal bovine serum (FBS; Gibco BRL, Grand Island, NY). T.T. was maintained in a 1:1 mixture of Dulbecco’s modified Eagle’s medium (DMEM; Nissui Pharmaceutical, Tokyo, Japan) and Ham’s F12 medium with 10% FBS. KYSE30 and KYSE50 were maintained in DMEM with 10% FBS. KYSE70 was maintained in DMEM with 2% FBS. KYSE150 was maintained in Ham’s F12 with 2% FBS.

### Patients

This study was performed retrospectively. The criteria for eligibility were histologically confirmed ESCC, surgery for stage I–III disease, absence of prior radiotherapy or chemo-therapy before surgery and the availability of a FFPE sample. Tumor specimens were collected from 246 patients with ESCC who were treated at the Kinki University Faculty of Medicine between 2001 and 2011. One sample was excluded because of poor DNA quality and 245 ESCC samples were finally evaluated. The World Health Organization Classification of Tumors was used for histologically grading. The tumors were staged according to the tumor-node-metastasis (TNM) classification of the American Joint Committee on Cancer (AJCC)/Union for International Cancer Control (UICC). The present study was approved by the institutional review board of the Kinki University Faculty of Medicine.

### Isolation of genomic DNA

Macro-dissection of the surgical specimens preserved as FFPE tissues was performed after deparaffinization to select a region of cancer tissue. Genomic DNA samples were extracted using a QIAamp DNA Micro Kit (Qiagen, Hilden, Germany) according to the manufacturer’s instructions. The DNA concentration was determined using the NanoDrop2000 (Thermo Scientific, Waltham, MA).

### Copy number assay

The DNA copy numbers of *EGFR, HER2, FGFR2* and *MET* were determined using commercially available and pre-designed TaqMan Copy Number Assays (Applied Biosystems, Foster City, CA), as described previously (15). The primer IDs used in this study were as follows: *EGFR*, Hs00997424_cn; *HER2*, Hs05475431_cn; *FGFR2*, HS05182482_cn (introns 14 and 15); and *MET*, Hs05005660_cn (introns 16 and 17). The TERT locus was used for the internal reference copy number. Human genomic DNA (Takara, Otsu, Japan) and DNA from non-cancer FFPE tissue were used as normal controls. The PCR analysis was performed using the ABI PRISM 7900HT Sequence Detection System (Applied Biosystems) and the results were analyzed using SDS 2.2 and CopyCaller software (Applied Biosystems).

### FISH analysis

FISH analysis of *EGFR* and *HER2* amplification was performed using the Vysis EGFR/CEP7 FISH Probe Kit (Abbott Laboratories, Abbott Park, IL) or the PathVysions HER2 DNA Probe Kit (Abbott Laboratories), according to the manufacturer’s instructions. Amplification was determined based on a *HER2/CEP17* signal ratio of >2.2. A two or more increase in the *EGFR* gene signal relative to the *CEP7* signal was considered to indicate gene amplification. The *FGFR2*-FISH method has been previously described (15), as has the *MET*-FISH method (18).

### Detection of EGFR mutations

EGFR mutations (exons 18–21) were detected using the Therascreen RGQ PCR kit (Qiagen), which combines Scorpions technology and the amplified refractory mutation system (ARMS) to detect mutations using real-time PCR. This sensitive method can detect 29 types of active mutations in the *EGFR* gene. All the reactions were performed according to the manufacturer’s instructions, as previously described (19).

### Cell growth inhibitory assay

To evaluate growth inhibition in the presence of various concentrations of EGFR-TKI AG1478 (Sigma), we used an MTT assay and a previously described method (20). Briefly, the cells were seeded at a density of 2×10^3^ cells/well in 96-well plates. Twenty-four hours later, AG1478 was added and the incubation was further continued for 72 h at 37°C. The assay was conducted in triplicate.

### Immunoblotting

A western blot analysis was performed as described previously (21). The following antibodies were used: polyclonal EGFR antibody, polyclonal phospho-EGFR antibody, polyclonal HER2 antibody, monoclonal HER4 antibody, polyclonal Akt antibody, monoclonal phosphor-Akt antibody, polyclonal p44/42 MAPK antibody, polyclonal phosphop-44/42 MAPK antibody, β-actin antibody and HRP-conjugated secondary antibody (Cell Signaling Technology, Beverly, MA); and monoclonal HER3 antibody (Upstate Biotechnology, Lake Placid, NY). The cells were cultured overnight in serum-starved medium and exposed to 0.1–10 *μ*mol/l of AG1478 for 3 h before the addition of EGF (10 ng/ml) for 15 min.

### Comparative genomic hybridization (CGH) analysis

The CGH analysis was performed using a SurePrint G3 Human CGH Microarray (Agilent Technologies, Santa Clara, CA) according to the manufacturer’s instructions. For the analysis, 0.2 *μ*g of DNA was extracted from each FFPE sample of ESCC and an *FGFR2*-amplified tumor or a non-cancer tissue were used as a control. The copy number changes were analyzed using Partek Genomic Suite 6.4 software (Partek Inc., St. Louis, MO).

### Statistical analysis

The prognostic analyses of the clinicopathological features and molecular factors were performed using a Cox regression. In the multivariate Cox models, the variable selection was based on the presence of significance (P<0.10) in a univariate analysis; variables that were not significant in the final model were removed using the stepwise method. The disease-free survival (DFS) and overall survival (OS) curves were constructed using the Kaplan-Meier method and were compared using the log-rank test. Statistical analyses were performed using PAWS Statistics 18 (SPSS Japan Inc., Tokyo, Japan).

## Results

### Patient results

Of the 245 patients evaluated in this study, all the patients had undergone surgery for histologically confirmed stage I–III ESCC. The patient characteristics are shown in Table I. The percentages of the pathological stages were as follows: stage I, 24%; stage II, 27%; and stage III, 49%. Fourteen (6%) patients had residual cancer at the time of surgery and tumor recurrence occurred in 98 (42%) patients. The median follow-up period was 24 months (range 0–126 months).

### Gene amplification of EGFR and HER2 in ESCC

**Figure 1:**
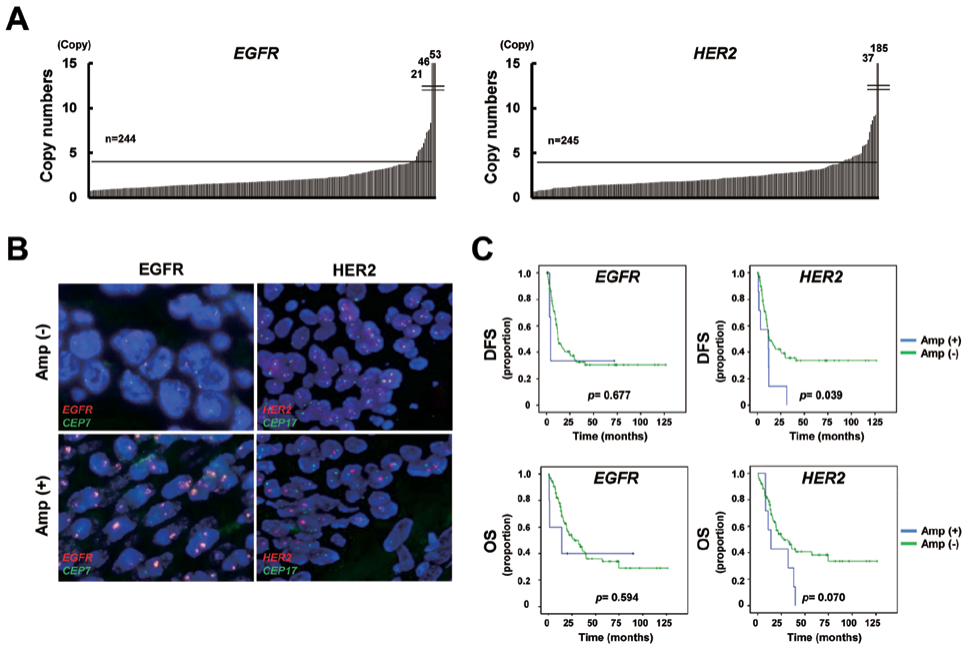
*EGFR* and *HER2* amplification in 245 esophageal squamous cell carcinoma (ESCC) specimens. (A) A TaqMan copy number assay was used to determine the DNA copy numbers of *EGFR* and *HER2*. DNA was extracted from formalin-fixed, paraffin-embedded samples. DNA copy numbers of >15 copies are shown on the bars. The *EGFR* copy number was not determined in one sample (n=244). Gene amplification of *EGFR* and *HER2* was observed in 7% (16/244) and 11% (27/245) of the ESCC specimens, respectively. (B) Fluorescence *in situ* hybridization analysis of *EGFR*-amplified or *HER2*-amplified ESCC specimens. Green, signal of *CEP7* or *CEP17* locus; red, signal of *EGFR* or *HER2* locus; amp, gene amplification. (C) Kaplan-Meier curves for disease-free survival (DFS) and overall survival (OS) for patients with stage III ESCC. Amp, gene amplification. The P-values were calculated using the log-rank test.

To determine the gene amplification of *EGFR* and *HER2* in FFPE samples, we used a high-throughput and real-time PCR-based copy number assay, as previously reported (15). Gene amplification was defined as more than four copies. The copy number assay showed that *EGFR* and *HER2* were amplified in 7% (16/244, one not determined; range 0.6–52.8 copies) and 11% (27/245; range 0.4–185.0 copies) of the ESCC specimens, respectively (Fig. 1A). FISH analysis demonstrated that the *EGFR*/CEP7 signal ratio was increased in *EGFR*-amplified samples, while the ratio was not increased in a non-amplified sample (Fig. 1B). Similarly, the *HER2/CEP17* signal ratio was consistent with the results of a copy number assay for *HER2*. FISH analysis verified the results of the copy number assays for *EGFR* and *HER2*.

### Prognostic impact of clinicopathological and gene amplification in ESCC

Of the 121 patients with stage III ESCC, 14 were excluded because of residual cancer and three were excluded because of the lack of copy number results; finally, 104 patients with stage III ESCC were evaluated to determine the prognostic impact of post-operative ESCC findings. The correlations between clinicopathological features, including age, sex, pathological tumor stage, pathological lymph node stage, tumor differentiation, lymphatic vessel invasion (Ly), vascular invasion (V) and the gene amplification statuses of *EGFR* and *HER2* and the DFS or OS were evaluated. A univariate analysis showed that the pathological lymph node stage, Ly grade, V grade and *HER2* amplification status were significant predictors of a poor DFS (Table II). A multivariate analysis revealed that the pathological lymph node stage (P=0.00003) and *HER2* amplification (P=0.021) were significant predictors of a poor DFS. Meanwhile, the pathological lymph node stage, tumor differentiation, Ly grade and V grade were significant predictors of a poor OS. A multivariate analysis demonstrated that the pathological lymph node stage (P=0.004) was a significant predictor of a poor OS. The Kaplan-Meier curves for DFS and OS plotted according to the gene amplification status are shown in Fig. 1C. These results indicated that *HER2* amplification, but not *EGFR* amplification, was a predictor of a poor outcome among postoperative patients with stage III ESCC in the present study.

### Active EGFR mutation in ESCC cell lines and clinical samples

**Figure 2:**
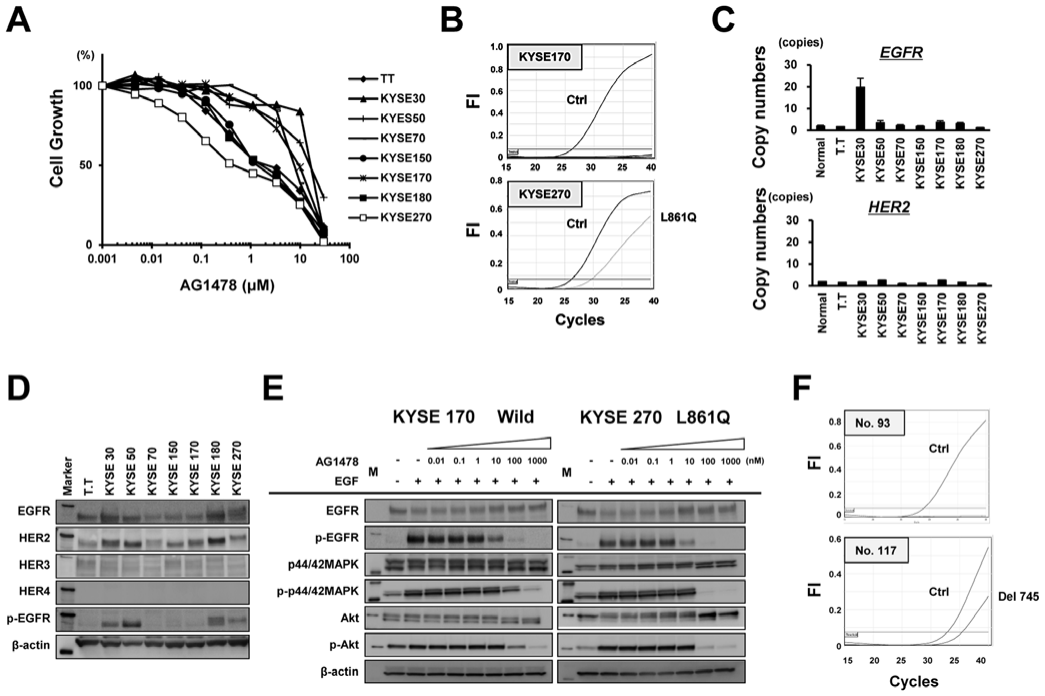
*EGFR* mutation in esophageal squamous cell carcinoma (ESCC) cell lines and clinical samples. (A) Growth inhibition in response to the EGFR tyrosine kinase inhibitor AG1478 was evaluated at the indicated concentrations using an MTT assay. (B) The status of the 29 types of *EGFR* mutation determined using the Scorpion-ARMS method in eight ESCC cell lines. Notably, KYSE270 cells, which were hypersensitive to AG1478, harbored the L861Q type of *EGFR* mutation, whereas the other cell lines did not exhibit any *EGFR* mutations. (C) The TaqMan copy number assay was used to determine the copy numbers of *EGFR* and *HER2* in ESCC cell lines. (D and E) Western blot analysis for EGFR, HER2, HER3, HER4 and phospho-EGFR expression in ESCC cell lines. β-actin was used as an internal control. Marker, molecular marker. Western blot analysis for expression levels of EGFR, phospho-EGFR, MAPK, phospho-MAPK, AKT and phospho-AKT in KYSE270 cells (L861Q) and KYSE170 cells (*EGFR* wild-type). The cells were exposed to AG1478 at the indicated concentrations for 3 h and then were stimulated with 10 ng/ml of EGF. β-actin was used as an internal control. M, molecular marker. (F) Among the 107 clinical ESCC samples that were evaluated, one (no. 117) carried a del745-750 type *EGFR* mutation.

We next examined the growth inhibitory effect of the EGFR-TKI AG1478 against eight ESCC cell lines to evaluate the effect of EGFR-TKI treatment on ESCC. Notably, the KYSE270 cells were hypersensitive to AG1478 at a sub-micro molar level of IC_50_ (0.45 *μ*M), which is similar to the hypersensitivity of lung cancer cells harboring an *EGFR* mutation (Fig. 2A). The possible presence of 29 types of *EGFR* mutations in the eight ESCC cell lines was examined using the Scorpion-ARMS method. The KYSE270 cells, which exhibited hypersensitivity to AG1478, harbored the L861Q type of *EGFR* mutation, whereas the other cell lines carried no mutations (Fig. 2B). A copy number assay revealed that *EGFR* was amplified in KYSE30 cells, while no significant amplifications of *HER2* were observed (Fig. 2C). The western blot analysis showed no significant overexpression of *HER2*, *HER3* or *HER4* (Fig. 2D), compared with a positive control (data not shown). The phosphorylation and protein expression levels of EGFR were increased in KYSE30, KYSE50, KYSE180 and KYSE270 cells. In the KYSE270 cells (L861Q), AG1478 completely inhibited the phosphorylation levels of MAPK, AKT and EGFR at a concentration of 100 nM, while phosphorylation was not inhibited in the KYSE170 cells (wild-type) at this concentration (Fig. 2E). These results indicate that an active *EGFR* mutation conferring hypersensitivity to EGFR-TKI was found in an ESCC cell line. Finally, we examined the presence of *EGFR* mutations in 107 clinical samples of ESCC. One ESCC tumor exhibited a del745-750 type of *EGFR* mutation (Fig. 2F). Thus, although the frequency of *EGFR* mutation was not high compared with NSCLC, we did find a mutation in a cell line and in a clinical sample of ESCC.

### Gene amplification of FGFR2 and MET in ESCC

**Figure 3:**
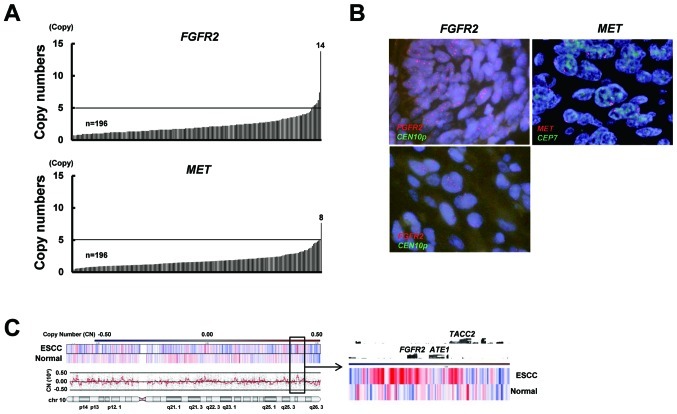
Gene amplification of *FGFR2* and *MET* in 196 esophageal squamous cell carcinoma (ESCC) specimens. (A) The TaqMan copy number assay was used to determine the copy number of *FGFR2* or MET. DNA was extracted from formalin-fixed, paraffin-embedded samples. The highest DNA copy numbers are shown on the bars. (B) Fluorescence *in situ* hybridization analysis of *FGFR2*-amplified or *MET*-amplified clinical samples. Green, signal of *CEN10p* or *CEP7* locus; red, signal of *FGFR2* or *MET* locus. (C) A comparative genomic hybridization analysis was performed using *FGFR2*-amplified ESCC tissue and non-cancer esophageal tissue (normal). The DNA copy number of chromosome 10 (left panel) and an enlarged amplified region (right panel) are shown.

To gain insight into molecular therapy targeting *FGFR2* or *MET* amplification in ESCC, we evaluated the amplifications of these genes. *FGFR2* and *MET* were amplified in 4% (8/196; range 0.4–13.8 copies) and 1% (2/196; range 0.4–7.7 copies) of the ESCC specimens, respectively (Fig. 3A). A FISH analysis confirmed the *FGFR2* and *MET* amplification (Fig. 3B). A CGH analysis showed that the *FGFR2* locus was amplified in *FGFR2*-amplified ESCC and that the amplicon seemed to consist of a relatively narrow region (Fig. 3C). The clinical features of *FGFR2*-amplified or *MET*-amplified ESCC are shown in Table III. Although the numbers of amplified cases were relatively small and, accordingly, definitive evidence could not be obtained, patients who had tumors with *FGFR2* or *MET* amplification seemed to have no significant trends regarding clinicopathological factors, including patient outcome. Collectively, these findings indicate that *FGFR2* amplification is present but that *MET* amplification is rare in ESCC.

## Discussion

*FGFR2* is frequently amplified in gastric cancer cell lines, especially in poorly differentiated type cells and amplification confers hypersensitivity to FGFR inhibitors (16,22). Regarding the mutation of *FGFR2*, somatic mutations of *FGFR2* have been found in 12% (15/122) of endometrial carcinomas and these *FGFR2* mutations have an oncogenic property that confers hypersensitivity to FGFR inhibitors (23). We demonstrated, for the first time, that *FGFR2* amplification was observed in ESCC. Our findings provide novel insight into FGFR-targeting therapy and further prospective studies evaluating *FGFR2* amplification in ESCC are needed. Meanwhile, recent study has demonstrated that 2% of patients (10/489) with esophagogastric adenocarcinoma harbored *MET* amplification and two of four patients with *MET*-amplified tumors treated with a *MET* inhibitor experienced tumor shrinkage (24). Although *MET* amplification is rare in ESCC, MET-targeted therapy may be a useful therapeutic approach in some cases.

The presence of active *EGFR* mutations or drug sensitivity to EGFR-TKI in ESCC cells remains unknown (25). We found that one out of eight ESCC cell lines harbored an L861Q mutation with hypersensitivity to EGFR-TKI and 1% (1/107) of clinical ESCC samples had a del745-750 type of mutation when examined using a highly sensitive detection method. In an *EGFR*-vIII-based method of overexpression, L861Q mutation reportedly enhances EGFR kinase activity and transforms activity without an increase in sensitivity to EGFR-TKI but with an increase in sensitivity to irreversible second-generation EGFR-TKI (26). Our results indicate that L861Q is an active mutation to EGFR-TKIs in ESCC cell lines; however, the 1% frequency of *EGFR* mutation in ESCC makes it difficult to stratify patients who may benefit from EGFR-TKI treatment, compared with NSCLC. For *HER2*-positive advanced gastric or gastroesophageal junction cancer, recent advances in the clinical development of molecular targeted therapy have enabled the use of trastuzumab as a standard therapy (27); however, similar regimens for the treatment of ESCC remain elusive. *EGFR* family-targeted therapy is considered to be the most promising approach to date, because EGFR and HER2 overexpression and amplification are frequently observed in ESCC. The anti-EGFR antibody cetuximab used in combination with radiotherapy or chemotherapy exhibited a significant clinical benefit when used against head and neck squamous cell carcinoma (28,29), leading to an ongoing intensive clinical trial using cetuximab for the treatment of ESCC. We detected *EGFR* and *HER2* amplification in 7 and 11% of ESCC specimens and our results support a rationale for introducing anti-EGFR and anti-HER2 antibody therapies to the treatment of patients with ESCC.

In conclusion, we determined the frequency of *EGFR, HER2, FGFR2* and *MET* amplification in ESCC and the presence of *EGFR* mutations among ESCC cell lines and clinical samples. Our results warrant serious consideration of the development of EGFR family inhibitors and FGFR-targeted therapies for ESCC exhibiting gene amplification.

## Figures and Tables

**Table I:** Patient characteristics.

Characteristics	No.
Age	
Range (years)	34 – 83
Median (years)	65
<60/≥60	55/190
Sex	
Male/female	208/37
Location	
Ut/Mt/Lt/Ae	20/149/68/8
pT	
T1/T2/T3/T4	69/45/124/7
pN	
N0/N1/N2/N3	89/77/50/29
pM	
M0/M1	245/0
pStage	
I/II/III/IV	59/65/121/0
Diff.	
Well/mod/por	48/141/56
Ly	
0/1	93/152
V	
0/1/2	205/40/0
Residual	
0/1/2	231/7/7
Recurrence	
(−)/(+)	133/98
Total	245

No., number of patients; diff., tumor differentiation; Ly, lymphatic vessel invasion; V, vascular invasion; residual, residual cancer; recurrence, recurrence of tumor had no residual cancer.

**Table II:** Univariate and multivariate analysis of clinical and molecular factors for disease-free and overall survival in stage III ESCC.

	Disease-free survival						Overall survival					
		
	Univariate analysis			Multivariate analysis			Univariate analysis			Multivariate analysis		
				
Factor	HR	95% CI	P-value	HR	95% CI	P-value	HR	95% CI	P-value	HR	95% CI	P-value
Age (≥60 vs.<60)	1.31	(0.75–2.30)	0.340				1.15	(0.66–2.01)	0.625			
Gender (male vs. female)	1.19	(0.56–2.49)	0.653				1.85	(0.80–4.33)	0.153			
pT (T3,T4 vs. T1, T2)	0.81	(0.45–1.47)	0.492				0.65	(0.36–1.17)	0.151			
pN (N3 vs. N1, N2)	3.44	(2.02–5.84)	0.000005	3.20	(1.85–5.53)	0.00003	2.81	(1.60–4.93)	0.0003	2.35	(1.32–4.20)	0.004
Diff. (por vs. well, mod)	1.49	(0.85–2.64)	0.168				2.10	(1.22–3.63)	0.008	1.70	(0.95–3.04)	0.092
Ly (1 vs. 0)	2.20	(1.16–4.18)	0.016	1.75	(0.81–3.82)	0.157	2.31	(1.15–4.64)	0.018	2.05	(0.89–4.74)	0.095
V (1, 2 vs. 0)	2.07	(1.21–3.53)	0.008	1.64	(0.95–2.82)	0.076	2.05	(1.20–3.53)	0.009	1.62	(0.92–2.85)	0.074
EGFR amp (+ vs. −)	1.39	(0.34–5.70)	0.650				1.40	(0.44–4.48)	0.575			
HER2 amp (+ vs. −)	2.31	(1.05–5.09)	0.038	2.59	(1.16–5.81)	0.021	2.15	(0.97–4.75)	0.060	2.05	(0.92–4.60)	0.081

Diff., tumor differentiation; Ly, lymphatic vessel invasion; V, vascular invasion; amp, gene amplification. Hazard ratios and 95% confidence intervals were computed using Cox regression.

**Table III:** Clinical features of *FGFR2*-amplified or MET-amplified ESCC.

No.	Age	Sex	Location	Macroscopic type	pT	pN	pM	pStage	Ly	V	Histology	Rec.	Rec. sites	*FGFR2* copies	*MET* copies	*FGFR2*	*MET*
1	79	M	Mt	3	T1b	N0	M0	I A	0	0	Mode	(−)		13.8	3.2	Amp	n
2	68	M	Mt-Lt	0-IIa	T1b	N0	M0	I A	0	0	Por	(−)		7.4	4.9	Amp	n
3	79	M	Mt	0-IIc	T1b	N0	M0	I A	0	0	Well	(−)		5.4	2.8	Amp	n
4	69	M	Mt	0-IIc+IIa	T2	N0	M0	IB	0	0	Mod	(−)		5.5	1.9	Amp	n
5	67	M	Lt	1	T2	N1	M0	II B	0	0	Por	(−)		5.7	0.7	Amp	n
6	71	M	Mt	0-I	T1b	N2	M0	III A	0	0	Mod	(−)		5.2	1.2	Amp	n
7	64	M	Lt	3	T3	N1	M0	III A	0	0	Well	(+)	Liver	5.0	3.8	Amp	n
8	63	M	Mt	2	T3	N2	M0	III B	0	1	Mod	(+)	Adrenal	6.2	2.4	Amp	n
9	63	F	Mt	1	T3	N0	M0	II A	0	0	Mod	(−)		3.0	7.7	n	Amp
10	75	M	Mt	0-IIc+IIa	T1b	N2	M0	III A	0	0	Mod	(+)	Lung	2.4	5.0	n	Amp

Location, tumor location in esophagus; well, well-differentiated; Ly, lymphatic vessel invasion; V, vascular invasion; amp, gene amplification. Macroscopic type, classification is based on the definitions of the Japanese Research Society for Gastric Cancer.
